# Improving protein function prediction by learning and integrating representations of protein sequences and function labels

**DOI:** 10.1093/bioadv/vbae120

**Published:** 2024-08-17

**Authors:** Frimpong Boadu, Jianlin Cheng

**Affiliations:** Department of Electrical Engineering and Computer Science, NextGen Precision Health Institute, University of Missouri, Columbia, MO 65211, United States; Department of Electrical Engineering and Computer Science, NextGen Precision Health Institute, University of Missouri, Columbia, MO 65211, United States

## Abstract

**Motivation:**

As fewer than 1% of proteins have protein function information determined experimentally, computationally predicting the function of proteins is critical for obtaining functional information for most proteins and has been a major challenge in protein bioinformatics. Despite the significant progress made in protein function prediction by the community in the last decade, the general accuracy of protein function prediction is still not high, particularly for rare function terms associated with few proteins in the protein function annotation database such as the UniProt.

**Results:**

We introduce TransFew, a new transformer model, to learn the representations of both protein sequences and function labels [Gene Ontology (GO) terms] to predict the function of proteins. TransFew leverages a large pre-trained protein language model (ESM2-t48) to learn function-relevant representations of proteins from raw protein sequences and uses a biological natural language model (BioBert) and a graph convolutional neural network-based autoencoder to generate semantic representations of GO terms from their textual definition and hierarchical relationships, which are combined together to predict protein function via the cross-attention. Integrating the protein sequence and label representations not only enhances overall function prediction accuracy, but delivers a robust performance of predicting rare function terms with limited annotations by facilitating annotation transfer between GO terms.

**Availability and implementation:**

https://github.com/BioinfoMachineLearning/TransFew.

## 1 Introduction

Proteins are essential molecules that play critical functional roles in biological systems. Their functions encompass catalyzing biochemical reactions, serving as structural elements, transducing cellular signals, defending against viruses, regulating gene activities, among others. Elucidating protein functions is crucial for gaining valuable insights into the molecular intricacies of biological systems. However, experimentally determining protein function is a time consuming and laborious process. Currently, fewer than 1% known proteins have function information determined experimentally according to the statistics in UniProt Consortium (2019). Therefore, it is important to develop computational methods to predict protein function from sequence and other relevant information.

In the realm of protein function prediction, there are two common challenges: (i) effectively integrating diverse information sources, such as protein sequence, protein-protein interaction, structural features, domain features, and biological texts, to accurately predict protein functions (Boadu *et al.* 2023), and (ii) accurately assigning rare or novel Gene Ontology(GO) terms (labels) (Aleksander *et al.* 2023) with few/no observations in labeled protein function datasets to new proteins that may have the function. It is harder to predict rare (low-frequency) GO terms than common GO terms because the former is less represented than the latter in the function datasets. But it is important to predict rare GO terms because they are usually specific and highly informative function classes that are more useful for generating biological hypotheses than common ones. Moreover, a large portion of all the GO terms are rather rare. Out of over 40 000 GO terms in the three main Gene Ontology categories: Cellular Component (CC), Molecular Function (MF), and Biological Process (BP), around 20 000 terms each are assigned to fewer than 100 proteins experimentally (Kulmanov and Hoehndorf 2022). Therefore, there is an urgent need to develop computational methods to predict rare function terms for proteins whose function is described by them.

Predicting rare GO terms is analogous to the few-shot learning problems (Sung *et al.* 2018) in various domains like computer vision (Dhakal *et al.* 2023, Safavigerdini *et al.* 2023, Giri and Cheng 2024), and natural language processing(NLP). For example, in the classification task of named entity typing (Yuan and Downey 2018, Zhang *et al.* 2020) in NLP, assigning rare entity types to entity names pose a similar challenge, due to the increasing size and granularity of entity types. Two kinds of methods, i.e. embedding-based methods and generative methods, have been proposed to tackle this challenge (Pourpanah *et al.* 2022). Embedding-based methods focus on learning an embedding space associating low-level features of highly annotated classes with semantic information of both highly annotated classes and rarely annotated classes to transfer knowledge from highly annotated classes to rarely annotated ones with few annotations. Generative methods generate features for rare classes based on samples from adequately annotated classes, converting the problem into the conventional supervised learning. In the protein function prediction, the hierarchical structure and textual descriptions of GO terms (classes/labels) provides us with the vital semantic information to transfer knowledge from the well-annotated classes to the ones with few or no annotations (Cao and Shen 2021).

In this study, we introduce an embedding-based deep learning method called TransFew to predict protein functions (Boadu *et al.* 2023), with an emphasis on improving the prediction of protein function described by rare GO terms. TransFew generates a function-relevant representations of a single protein sequence in the sequence space using a pretrained protein language model [i.e. ESM2 (Lin *et al.* 2022, Boadu *et al.* 2023)] and multi-layer perceptrons (MLP). The sequence representation of a protein is generated by multiple MLP modules with residual connections each designed to predict functions for proteins in terms of a specific group of GO terms with similar annotation frequency, which therefore cover all the GO terms from rare ones to common ones equally. TransFew also generates a semantic representation of all the GO terms (labels) in the label space from their textual description (definition) and their hierarchical relationships in the Gene Ontology graphs [e.g. the inheritance and composition relationships (i.e. similarity) between GO terms] using a graph convolutional neural network (GCN)-based auto-encoder and a biological natural language model (BioBert) (Lee *et al.* 2020), which facilitates the transfer of annotations from common GO terms to rare ones according to their relationships. TransFew then uses a joint feature label embedding technique based on the cross attention to integrate the label representations and sequence representations to accurately predict protein functions.

TransFew not only improves the overall accuracy of protein function prediction, but also is robust against the low frequency of rare GO terms.

## 2 Methods

**Figure 1.**
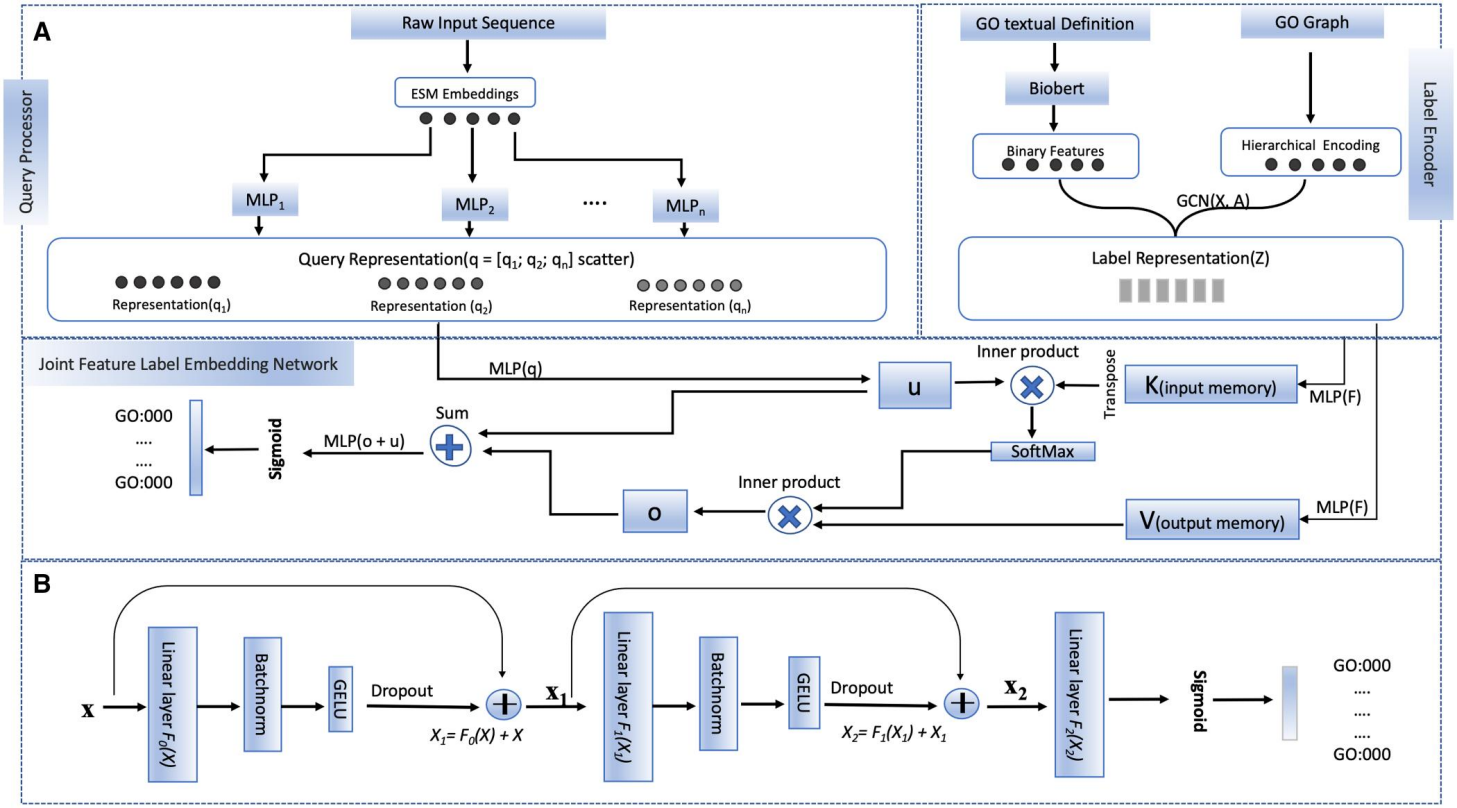
The overall architecture of TransFew. (A) The three components of TransFew: a query processor (top left) to generate sequence representation using multi-layer perceptrons (MLPs) and ESM2, a label encoder (top right) to extract label representations using Biobert (Lee *et al.* 2020) and a graph convolutional neural networks (GCN)-based auto-encoder (Kipf and Welling 2016), and a joint feature-label embedding network to combine sequence and label representations via the cross attention for a MLP to make final function prediction. (B) The detailed design of a typical MLP module used in TransFew.

The overall architecture of TransFew is illustrated in Fig. 1. It has three components: (i) a query processor consisting of multiple MLPs to extract function-relevant sequence representations from a protein sequence (query), (ii) a label encoder to extract label representations for all the GO terms (labels), and (iii) a joint feature-label embedding network to combine sequence and label representations to predict the function of a protein. One TransFew model was trained to predict the GO terms in each of the three GO function categories [molecular function (MF), cellular component (CC), and biological process (BP)], respectively.

### 2.1 Query processor

The query processor is to generate the function-relevant sequence representations for proteins. Protein function terms have very different annotation frequency in the labeled protein function datasets. Here, the annotation frequency of a GO term is the number of proteins that are labeled to have it as function. Rare GO terms are the ones that only occur to be the function labels of a small number of proteins. Generating a simple representation for all the GO terms by one MLP regardless of their frequency allows the common GO terms to dominate the rare (low-frequency) GO terms, which can reduce the accuracy of predicting them. Therefore, we partitioned GO terms into *n* groups for a Gene Ontology category (i.e. MF, CC, or BP) based on their annotation frequency, and designed *n* MLPs to collectively learn representations for proteins across all GO terms. These MLPs function as multiple experts, each generates a representation for an input protein whose dimension is equal to the number of GO terms in one group. Like inception networks (Szegedy *et al.* 2016), the different number of output dimensions can facilitate the MLPs to learn different aspects of GO terms, but it is not guaranteed that which group of GO terms are learnt by which MLP. Instead, the concatenation of the representations of all the MLPs generates a full GO representation for the input protein whose dimension is equal to the total number of GO terms. Specifically, the GO terms of BP were partitioned into three groups and the GO terms of CC and MF into two groups. The statistics for the partitions is shown in Supplementary Table S1.

Each MLP (i.e. MLP_*i*_) takes as input the sequence features of a protein generated by a large pretrained protein language model, ESM2_t48 (Lin *et al.* 2022) from its sequence, and outputs a vector $q_{i}\in R^{D_{\left||G_{i}|\right|}}$, where $\left||G_{i}|\right|$ is the number of GO terms in a GO group *G_i_*. ESM-2t48 (Lin *et al.* 2022) accepts the sequence of a protein as input and generates feature embeddings at multiple layers. Here, the per-residue embeddings of the last layer (48th layers) are taken out and averaged by the mean aggregator to generate the embedding of the protein, whose dimension is 5120. For a protein sequence exceeding the length limit of ESM2_t48, i.e. 1022 residues, it is divided into chunks of length 1022 except the last chuck that may have fewer than 1022 residues, each of which is processed by ESM2_t48 separately. The embeddings for all the chunks are concatenated as the embedding of the full protein sequence. In addition to using ESM-2t48 to generate input features for the MLP, we also tried to use multiple sequence alignments (MSAs) (Rao *et al.* 2021) and InterPro domain annotations (Yao *et al.* 2021, Kulmanov and Hoehndorf 2022, Paysan-Lafosse *et al.* 2023, Wang *et al.* 2023) of proteins to generate input features for the MLPs. The details of generating MSAs and InterPro domain annotations are described in Supplementary Notes S1 and S2. However, according to the ablation study, adding them on top of the features based on ESM-2t48 does not improve protein function prediction accuracy, and therefore they are not included into the final version of TransFew.

The detailed architecture of a MLP of generating the representation of a protein from its sequence features is depicted in Fig. 1B. The MLP has multiple blocks, each of which has a fully connected linear layer, followed by a batch normalization layer and a Gaussian Error Linear Unit (GELU). The input for each block except for the last one is added to its output via a skip connection, resulting in a residual network. The output of the last block is used as input for a sigmoid function to predict the probability of each GO term represented as logit.

The entire query processor, along with all other components, is jointly trained. The output *q_i_* (a vector of predicted logits whose dimension is equal to the number of GO terms in a group *G_i_*) generated by each MLP for an input protein are combined to form the final semantic representation of the protein across all GO terms in a gene ontology (MF, CC, or BP). This integrated representation serves as the output of the query processor. The combination process involves employing a scatter operation (Paszke *et al.* 2017), wherein the values produced by each MLP are distributed within the query representation tensor to match the predefined order of the GO terms.

### 2.2 Label encoder

The label encoder in Fig. 1A is used to generate semantic representations for all the GO terms (labels) under consideration. Two types of label data, i.e. the relations between GO terms in a GO Graph and the definition of GO terms (the textual descriptions) are used as input for the label encoder.

The relationships between GO terms (nodes) in a GO graph are represented by an adjacency matrix *A*, where each row encodes the relationships of a node. The entry *A_ij_* is set to 1 if node *i* is an ancestor of *j* or equal to *j*, and 0 otherwise. *A* encodes the hierarchical relationships between the GO terms.

For the definitions of the GO terms, we collected the textual description of each GO term, which contains what the term represents as well as reference(s) to the original source of the information. The textual description of each GO term is used by a pre-trained biomedical language model, BioBert (Devlin *et al.* 2018, Lee *et al.* 2020), to generate an embedding for it. The dimension of the embedding (*D_e_*) is 768, which is set by BioBERT. The embedding is considered the semantic features of each GO term.

The hierarchical relationships and the semantic embeddings of the GO terms are integrated by a graph auto-encoder model (Kipf and Welling 2016) to generate the representation of all the GO terms (labels). The input for the model is a GO graph, in which the relationships between nodes (GO terms) are stored in the matrix *A* and the feature of each node is its semantic embedding generated from the textual description of its GO term. The model uses an encoder-decoder architecture, where the encoder is a two-layer graph convolutional network (GCN) (Kipf and Welling 2017) defined as:

(1) $$\begin{array}{c} \mathrm{GCN}(X,A)=\tilde{A}R\mathrm{eLU}(\tilde{A}XW_{0})W_{1}\\ \tilde{A}=D^{-\frac{1}{2}}AD^{-\frac{1}{2}} \end{array}$$

*W*_1_ and *W*_2_ are the weight matrices, $\tilde{A}$ is the symmetrically normalized form of the *A*, and *X* is the matrix of the semantic embeddings of all the GO terms. *ReLU* denotes the ReLU activation function. We use the inner product decoder to reconstruct *A* as $\hat{A}$ from the embeddings *Z* outputted by the GCN model as follows:

(2) $$\begin{array}{c} Z=\mathrm{GCN}(X,A)\;\text{and}\;\hat{A}=\sigma (ZZ^{⊤}) \end{array}$$

where σ(·) is the logistic sigmoid function. The graph auto-encoder model was pretrained to reconstruct the GO Graph, *A*, from *A* itself and the semantic embeddings of the GO terms, through the self-supervised learning. After the training, the *Z* ($Z\in R^{D_{c}\times D_{e}}$) extracted from the bottleneck layer of the GCN-based autoencoder is used as the label representation, where *D_c_* is the number of GO terms and *D_e_* represents the dimension of the label representation (in this work *D_e_* = 1024). It is worth noting that the label representation is independent of any protein.

In addition, we investigated two alternative encoder architectures, such as Graph Attention Networks (GAT) (Veličković *et al.* 2017) and Graph Transformer (TransformerConv) (Shi *et al.* 2020) to combine the features of the textual description and GO term relationships, but they did not perform better than the GCN-based auto-encoder (see Supplementary Note S4).

### 2.3 Joint feature-label embedding network

We developed a joint feature-label embedding network to fuse the sequence representation of a protein generated by the query processor with the label representation of all GO terms generated by the pretrained label encoder to predict the protein’s associated GO terms (Fig. 1A). In contrast to prior approaches, such as the use of a bilinear function (Xu and Wang 2022) or a scoring function based on softmax and 1D convolutional networks (Cao and Shen 2021), we introduced a cross attention-based joint embedding model. This model facilitates the projection of the protein representation and the label representation into a shared embedding space, enhancing protein function prediction accuracy.

Given the label representation $Z\in R^{D_{c}\times D_{e}}$, and the query protein representation $q\in R^{D_{c}}$, TransFew converts the query representation *q* to $u\in R^{D_{m}}$ using a linear layer as follows: $u=W_{q}^{⊤}q$, and $W_{q}\in R^{D_{c}\times D_{m}}$, and constructs two memory components: key $K\in R^{D_{c}\times D_{m}}$ and value $V\in R^{D_{c}\times D_{m}}$ from Z, using two embedding matrices $W_{k}\in R^{D_{b}\times D_{m}}$ and $W_{v}\in R^{D_{b}\times D_{m}}$ respectively. The cross attention between the representation of a query protein *q* and the representation of all the GO term ($g_{i}\in R^{D_{e}},\;i\in \{1,2,\ldots D_{c}\}$) is computed as:

(3) $$\begin{array}{c} \mathrm{Attention}(u,K,V)=\mathrm{softmax}(\frac{uK^{⊤}}{\sqrt{d_{k}}})V \end{array}$$

where *d_k_* = *D_m_*.

The representation of the query protein and the cross attention are combined by a MLP with a residual connection to predict the probability of GO terms (*y*) for the query protein as follows:

(4) $$\begin{array}{c} y=\mathrm{sigmoid}(W^{⊤}(o+u)) \end{array}$$

where $W\in R^{D_{c}\times D_{m}}$ and o=Attention(u,K,V)

The feature-label embedding network and the query processor of the model (Fig. 1A) were optimized by minimizing the binary cross-entropy loss between predictions and true labels during training, while the weights of the pretrained label encoder were kept fixed. It is worth noting that the protein function prediction problem is a multi-label classification problem, in which a protein may have multiple correct labels.

### 2.4 Datasets

We collected proteins from the UniProtKB/Swiss-Prot data repository that were released by November 2022 for training and validation. The proteins were split into the training dataset and test dataset according to the 90%–10% ratio. The functional annotations (GO terms) of the proteins were obtained from from UniProt, and the GO ontology graph as well as GO textual data were collected from the Gene Ontology Resource (Ashburner *et al.* 2000, Aleksander *et al.* 2023). To get all the terms (labels) associated with a protein, we first retrieved its immediate GO terms provided in UniProt and then for each immediate GO term we traveled up the GO graph to retrieve all its ancestor GO terms. Only the GO terms with relatively strong evidence codes: EXP, IDA, IPI, IMP, IGI, IEP, TAS, IC, HTP, HDA, HMP, HGI, HEP are used as the function labels for each protein, following the criteria used in the Critical Assessment of Functional Annotation (CAFA) (Radivojac *et al.* 2013).

To create an independent test dataset, we obtained proteins in the UniProtKB/Swiss-Prot database whose function annotation were released in December 2023. This test dataset is called Test_all. Moreover, we used MMseqs (Steinegger and Söding 2017) to filter out the sequences in Test_all that have >30% identity with the sequences in the training dataset to create a redundancy reduced dataset—Test_novel, which is used to test how well TransFew can generalize to new proteins that have little or no sequence similarity with the training proteins.

The number of proteins in the training dataset, validation dataset, Test_all dataset, and Test_novel dataset for each gene ontology category (MF, CC, and BP) is reported in Supplementary Table S2.

## 3 Results and discussions

### 3.1 Benchmarking TransFew with baseline methods on the test datasets

We compared TransFew with six other methods [Naive, DiamondBLAST (Kulmanov and Hoehndorf 2020), Tale (Cao and Shen 2021), NetGO 3.0 (Yao *et al.* 2021, Wang *et al.* 2023), DeepGO-SE (Kulmanov *et al.* 2023), and SPROF-GO (Yuan *et al.* 2023)] on the Test_all dataset in terms of multiple metrics of evaluating protein function prediction, including F_*max*_, area under the precision–recall curve (AUPR), weighted F_*max*_, and S_*min*_ of measuring the uncertain/missing information in function predictions (Clark and Radivojac 2013, Jiang *et al.* 2016, Zhou *et al.* 2019, Paolis 2023, Piovesan *et al.* 2024) (see the detailed definition of the evaluation metrics and summary of the baseline methods in Supplementary Notes S5 and S6 respectively).

**Figure 2.**
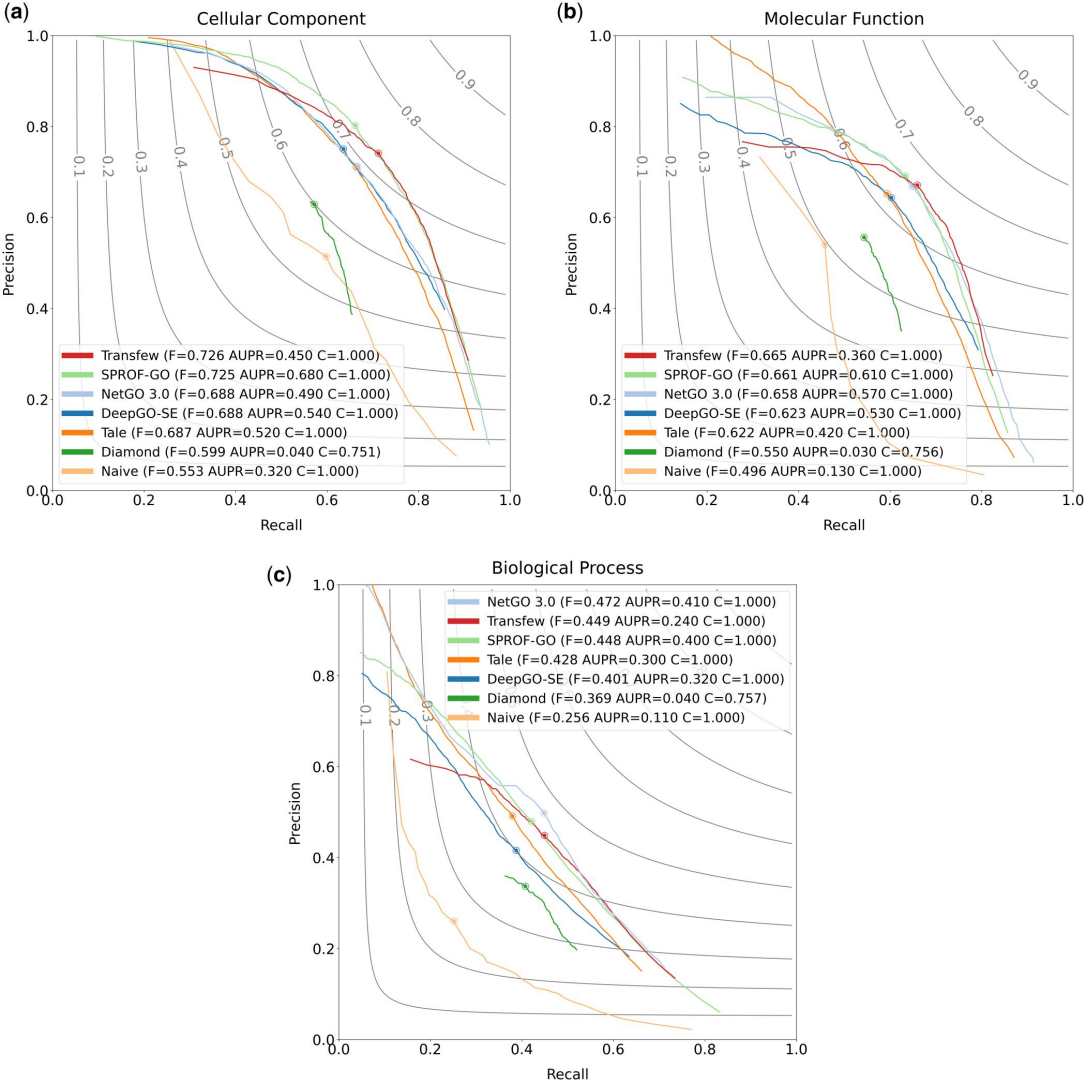
The precision–recall curves of TransFew, Naive, DiamondBLAST, Tale, NetGO 3.0, SPROF-GO, and DeepGO-SE for the three ontologies (BP, MF, and CC) on the Test_all dataset, respectively. The circled dot highlights the point where each method achieves the highest *F*_max_.

The results of TransFew, Naive, DiamondBLAST, Tale, NetGO 3.0, SPROF-GO, and DeepGO-SE on the Test_all dataset are presented in Table 1A. TransFew performs best in Cellular Component and Molecular Function, and ranks second in Biological Process in terms of F_max. In terms of weighted F_max, TransFew leads in Molecular Function and Biological Process, and comes second in Cellular Component. Lastly, in terms of S_min, TransFew achieves the second-best performance in Cellular Component and ranks third in Molecular Function and Biological Process. The precision–recall curves for the four methods across the three gene ontology categories (BP, MF, and CC) on the Test_all dataset are shown in Fig. 2. In terms of AUPR, TransFew performs better than Naive, DiamondBLAST but worse than the other deep learning/ensemble methods. The reason why TransFew has a lower AUPR score than the other deep learning methods is that its precision–recall curve covers a narrower range of recall than theirs (see Fig. 2).

**Table 1.** The performance of TransFew, Naive, DiamondBLAST, Tale, NetGO 3.0, SPROF-GO, and DeepGO-SE, on the test datasets in the three GO categories (BP, MF, and CC): (A) The results on all the new proteins in Test_all. (B) The results on Test_novel comprised proteins that have ≤30% sequence identity with the proteins in the training dataset of TransFew.^a^

(A) On Test_all												
Methods	*F_max_* (↑)			*WF_max_* (↑)			*AUPR* (↑)			*S_min_* (↓)		
	CC	MF	BP	CC	MF	BP	CC	MF	BP	CC	MF	BP
Naive	0.5528	0.4958	0.2558	0.3715	0.3400	0.2088	0.3216	0.1363	0.1100	9.3167	8.6241	22.2875
DiamondBLAST	0.5989	0.5496	0.3689	0.4864	0.4806	0.3275	0.0494	0.0388	0.0456	9.1097	9.7205	32.6331
Tale	0.6867	0.6215	0.4280	0.5559	0.5168	0.3711	0.5260	0.4291	0.3051	7.1850	6.9453	19.6918
NetGO 3.0	0.6885	0.6583	**0.4716**	0.5728	0.5730	0.4021	0.4969	0.5767	**0.4101**	6.8368	6.2940	19.2362
DeepGO-SE	0.6883	0.6227	0.4011	0.5334	0.5229	0.3569	0.5425	0.5336	0.3201	7.7345	6.9489	20.7057
SPROF-GO	0.7249	0.6612	0.4483	**0.6125**	0.5769	0.4022	**0.6869**	**0.6197**	0.4016	**6.2503**	**6.2124**	**19.1805**
TransFew	**0.7264**	**0.6655**	0.4489	0.6109	**0.5860**	**0.4067**	0.4546	0.3633	0.2442	6.6936	6.3848	19.3534

(B) On Test_novel												
Methods	*F_max_* (↑)			*WF_max_* (↑)			*AUPR* (↑)			*S_min_* (↓)		
	CC	MF	BP	CC	MF	BP	CC	MF	BP	CC	MF	BP
Naive	0.5464	0.5033	0.2545	0.3735	0.3523	0.2062	0.3080	0.1429	0.1083	9.4576	8.7214	22.4676
DiamondBLAST	0.5591	0.5096	0.3437	0.4478	0.4412	0.3026	0.0456	0.0373	0.0431	9.0348	9.3545	29.7683
Tale	0.6707	0.5994	0.4223	0.5387	0.4831	0.3668	0.5039	0.4041	0.3010	7.4662	7.4161	19.5395
NetGO 3.0	0.6769	0.6414	**0.4750**	0.5611	0.5465	**0.4022**	0.4938	0.5563	**0.4123**	6.9270	6.6866	**19.3370**
DeepGO-SE	0.6811	0.6096	0.4024	0.5290	0.5037	0.3569	0.5317	0.5101	0.3191	7.9617	7.2411	20.8680
SPROF-GO	0.7130	0.6449	0.4493	0.6004	0.5527	0.3999	**0.6774**	**0.5977**	0.4057	**6.4120**	**6.5325**	19.4221
TransFew	**0.7189**	**0.6476**	0.4433	**0.6024**	**0.5601**	0.4013	0.4486	0.3513	0.2574	6.7652	6.8034	19.4462

a Bold font highlights the best result. TransFew was trained using all the GO terms with at least one annotation in the training dataset.

On the Test_novel dataset consisting of proteins that have ≤30% sequence identity with the proteins in the training data, TransFew achieves the best performance in Cellular Component and Molecular Function categories in terms of two metrics: F_max, and weighted F_max scores. For Biological Process, it ranks second in terms of weighted F_max, and third in terms of F_max. In terms of S_min, TransFew ranks second in Cellular Component and third in Molecular Function and Biological Process(Table 1B). Similar to the performance on the Test_all dataset, Transfew has a higher AUPR score than Naive and DiamondBLAST but a lower AUPR score than the other deep learning/ensemble methods. The performance of TransFew on Test_novel is only moderately lower than on Test_all in terms of different metrics, indicating that it generalizes well to new test proteins that have no or little sequence identity with the training proteins.

It is worth noting that TransFew is a pure machine learning method, while other methods such as NetGO 3.0 and SPROF-GO combines machine learning predictions and homology-based function annotation transfer to make final prediction. The results show that the performance of a pure end-to-end machine learning method like TransFew is comparable to ensemble methods based on both machine learning and homology transfer for protein function prediction.

### 3.2 Performance of predicting rare GO terms

**Figure 3.**
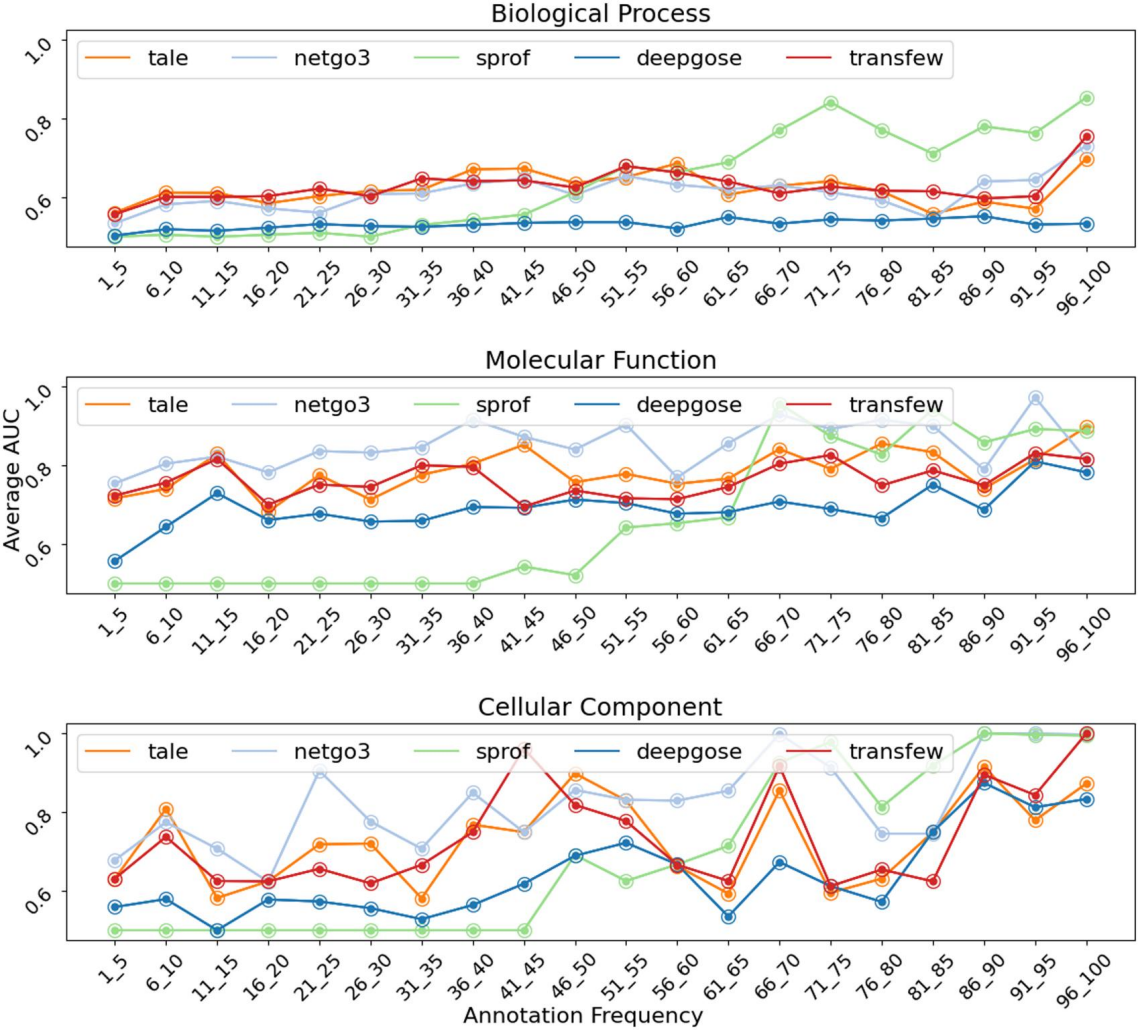
The average AUC score of five methods (TransFew, Tale, and NetGO 3.0, DeepGO-SE, and SPROF-GO) predicting relatively rare GO terms in each GO group within an interval of annotation frequency for the three gene ontologies (BP, MF, and CC). The *X*-axis represents the annotation frequency of the GO term groups. The *Y*-axis represents the average AUC scores.

We investigated how well TransFew predicts rare GO terms with low annotation frequency. GO terms with ≤100 annotations were grouped into 20 categories based on their number of annotations (frequency) in the training data, using an interval size of 5. Figure 3 shows the average AUC (area under ROC curve) scores of TransFew, Tale, NetGO 3.0, DeepGO-SE, and SPROF-GO for predicting GO terms in each group for BP, MF, and CC. For biological process, TransFew and Tale generally perform best for rare GO terms with ≤55 annotations, while SPROF-GO has the highest average AUC scores for GO terms with ≥60 annotations. For molecular function, NetGO 3.0 performs best for very rare GO terms with ≤40 annotations, followed by TransFew and GO. For cellular component, the overall AUC scores of TransFew are more or less ranked in the middle for GO terms with ≤40 annotations. The Pearson’s correlation between the AUC scores of TransFew and the annotation frequency of the GO terms in BP, MF and CC is only 0.42, 0.37, and 0.45, respectively. The moderate correlation indicates its performance is robust with the respect to the annotation frequency of rare GO terms.

### 3.3 The contributions of different components and implementations of TransFew

We tested how different components or implementations of TransFew influenced its performance. MLP (Interpro), MLP (MSA), and MLP (ESM) denote the three implementations of using the Interpro domain features, the MSA features, and the sequence features generated by ESM2_t48 respectively to generate the sequence representation for function prediction, without using the label representation at all. TransFew stands for the final implementation that combines the sequence representation generated from the ESM2_t48 features and the label representation to predict protein function. TransFew + MSA + Interpro is the same as TransFew except that it uses ESM2_t48 features together with the MSA and Interpro features to generate the sequence representation. MLP (Interpro), MLP (MSA), and MLP (ESM) were trained on the GO terms that have at least 30 annotations, while TransFew and TransFew + MSA + Interpro were trained on the GO terms with at least one annotation. The results of the different implementations are shown in Table 2.

**Table 2.** The performance of different components or implementations of TransFew on the Test_all dataset in the three GO categories (BP, MF, and CC).^a^

Methods	*F_max_* (↑)			*WF_max_* (↑)			*AUPR* (↑)			*S_min_* (↓)		
	CC	MF	BP	CC	MF	BP	CC	MF	BP	CC	MF	BP
Interpro	0.6753	0.6340	0.4283	0.5396	0.5424	0.3631	0.7485	0.6424	0.3880	7.0100	6.6078	19.7022
MSA	0.6931	0.6397	0.4167	0.5662	0.5542	0.3657	0.7689	0.6524	0.3769	6.8752	6.7176	19.8060
ESM	0.7113	0.6652	**0.4578**	0.6058	0.5769	**0.4165**	0.7960	0.6745	0.4344	**6.5683**	6.5698	**19.0910**
TransFew	**0.7264**	**0.6655**	0.4489	**0.6109**	**0.5860**	0.4067	0.7928	0.6871	**0.4392**	6.6935	**6.3848**	19.3534
TransFew+	0.7065	0.6426	0.3732	0.5871	0.5519	0.3055	**0.7982**	**0.7016**	0.3388	6.9579	6.7165	20.4570

a TransFew is the final version of the method in this work. Interpro, MSA, ESM, and TransFew+ stand for MLP (Interpro), MLP (MSA), MLP (ESM), and TransFew + MSA + Interpro, respectively. Bold numbers denote the best results.

Among the three methods of using only sequence representations to predict protein function, MLP (ESM) performs better than MLP (Interpro) and MLP (MSA) in terms of all the metrics for all three gene ontologies, indicating that the ESM2_t48 features are better than the MSA features and the Interpro features for generating sequence representations for protein function prediction.

TransFew that combines the sequence representation generated by ESM2_t48 and the label representation generated from the GO graph and the text description of GO terms performs better than MLP (ESM) without using the label representation in most situations, suggesting that integrating the sequence representation and the label representation can generally improve protein function prediction.

The combination of TransFew with additional features from MSA and Interpro outperforms TransFew in only two cases in terms of the AUPR metric, suggesting the combination does not improve the performance in this experiment. However, multiple studies (Kulmanov and Hoehndorf 2020, Yao *et al.* 2021, Wang *et al.* 2023, Yuan *et al.* 2023) have demonstrated that integrating features from diverse modalities typically enhances function prediction performance. We hypothesize that the decline in the performance in this experiment may stem from the addition of extra features not substantially increasing relevant information but rather adding complexity to the model, potentially leading to overfitting and a decrease in the generalization performance. Indeed, Supplementary Note S3 reveals that even though TransFew + MSA + Interpro fits the training data better than TransFew, it performs worse on the validation data. Therefore, we plan to explore more effective methods of integrating information from multiple modalities in the future.

Finally, we compared the performance of TransFew of using the label encoder with that of its other implementations that had the embedding of the label encoder replaced by a random matrix for the various GO partition groups in Supplementary Note S7. The results demonstrate that using the label encoder improves the prediction accuracy across the board.

## 4 Conclusion and future work

In this work, we present a new approach (TransFew) combining the information in the input space (the protein sequence space) and the output space (the function label space) to improve protein function prediction, particularly the accuracy of predicting rare function terms (GO term labels with few annotations). In the input space, we use a large pretrained protein language model to generate features for a protein sequence, which are then mapped to the protein function space defined by both common and rare GO terms to create a function-relevant representation for the protein. Learning the unbiased representations of proteins in terms of both common and rare GO terms makes it possible to predict them on the equal footing. In the output space, we use a graph convolutional neural network-based auto-encoder to combine the textual definition of GO terms and the inheritance and composition relationships between GO terms in the GO graphs to generate the semantic representations for all the GO terms, capturing the similarity between GO terms to facilitate the transfer of annotation from common GO terms to rare ones.

The representations in the input space and the output space are integrated by a novel cross-attention mechanism to build the associations between the protein representation and the label representation, which are used to predict the final function terms for the protein. TransFew not only performs better than two highly sophisticated protein function prediction methods on newly released test proteins, but also can predict the rare GO terms more accurately, demonstrating the approach of representing and combining the data of GO terms and proteins is effective in predicting all kinds of GO terms.

Our experiment also demonstrates that the sequence features generated by a large protein language model (ESM2_t48) is sufficient to create a functional-relevant representation of protein sequences that is more useful for protein function prediction than the features generated from multiple sequence alignments (MSAs) and Interpro domain features. Even though this does not rule out the usefulness of MSAs and Interpro domain features, it does show that very large pretrained protein language models can effectively capture the evolutionary patterns in protein sequences relevant to protein function prediction.

Because TransFew uses only a single protein sequence as input without the need of searching large protein sequence databases to generate multiple sequence alignments, it can predict protein function quickly, making it applicable to predicting the function of proteins in an entire proteome of a species.

Even though three modalities of data including protein sequences, textual description of GO terms, and hierarchical relationship between GO terms have been integrated by TransFew to predict protein function, other relevant modalities of protein data (Cao and Cheng 2016) such as protein structures, protein-protein interaction, hypothetical function annotations based on homology transfer, and the textual description of proteins have not be explored in this work. In the future, we plan to add all these modalities into our approach, harnessing the combined power of diverse features to further enhance the accuracy and robustness of protein function prediction. One promising avenue is to leverage multi-modal language models for protein function prediction, as demonstrated in computer vision (Radford *et al.* 2021, Acosta *et al.* 2022, Alayrac *et al.* 2022) and a recent bi-modal protein language model (Heinzinger *et al.* 2023). By adopting such an approach, we can potentially learn a comprehensive representation of proteins encompassing protein sequence, structure, interaction, gene ontologies, and prior human knowledge to improve protein function prediction.

## Supplementary Material

vbae120_Supplementary_Data

## Data Availability

The data underlying this article are available at BioinfoMachineLearning/TransFew: Transformer for protein function prediction (version 2) (github.com).
