# Supplementary material for: Improving protein function prediction by learning and integrating representations of protein sequences and function labels
Source: Bioinform Adv. 2024 Aug 17;4(1):vbae120. doi: 10.1093/bioadv/vbae120 (PMC11374024; doi:10.1093/bioadv/vbae120)
Supplement: vbae120_Supplementary_Data [file vbae120_supplementary_data.pdf]

IMPROVING PROTEIN FUNCTION PREDICTION BY LEARNING AND INTEGRATING REPRESENTATIONS OF PROTEIN SEQUENCES AND FUNCTION LABELS

–

Supplementary Materials

Author: Frimpong Boadu, Jianlin Cheng

Table S1: The partition of GO terms into groups according to their annotation frequency for three gene ontologies (BP, MF, and CC). MF and CC terms are partitioned into two groups according to the frequency threshold of 30 respectively, while BP terms are partitioned into three groups because BP has many more GO terms than MF and CC. The last column reports the number of GO terms in each partition.

**Ontology**	**Partition**	**Number of GO Terms**
BP	1 - 5	7892
BP	6 - 30	6977
BP	$>$ 30	6415
MF	1 - 30	6040
MF	$>$ 30	1183
CC	1 - 30	2083
CC	$>$ 30	873

Supplementary Note 1: Generating MSA for a protein

We use HHblits to create a multiple-sequence alignment (MSA) for a protein sequence from the $UniRef$30_2022_02 database [9]. Subsequently, we filter out lowercase letters and insertion characters like ".", and "*" from the alignment. Employing a greedy algorithm[12], we choose 128 sequences that maximize the Hamming distance within the MSA. Finally, we employ the pre-trained ESM-MSA-1b [12] language model to generate representative embeddings for the protein using its MSA. The embeddings are used as input for the various MLP sub-models to generate representative embeddings for the protein.

Supplementary Note 2: Generating Interpro domain features for a protein

We generate InterPro domain features from a protein sequence using InterProScan. Interpro features are represented as a binary matrix, where a 1 implies that a protein has an Interpro signature and 0

otherwise. In each of the three GO ontologies, we consider only signatures that appear in the training data, resulting in an input vector of dimension 24714, 25523 and 24846 for cellular component, molecular function and biological process respectively. The binary vector is then used as input for the various MLP sub-models to generate representative embeddings for the protein.

Supplementary Note 3: Compare the training and validation process of TransFew and TransFew + InterPro + MSA

Figure S1 compares how TransFew and TransFew + InterPro + MSA behaved in the training and validation processes.

Supplementary Note 4: Three label embedding methods

We explored three graph neural network-based auto-encoders to combine the features generated from the textual descriptions of GO terms using BioBERT and the ones from the hierarchical relationships between GO terms to create the representation of all GO terms, which are Graph Convolutional Network (GCN)-based auto-encoder[5], Graph Attention Networks (GAT)-based auto-encoder [1, 16], and Graph Transformer (TransformerConv)-based auto-encoder [14]. Their performance in the validation process is shown in Figure S2.

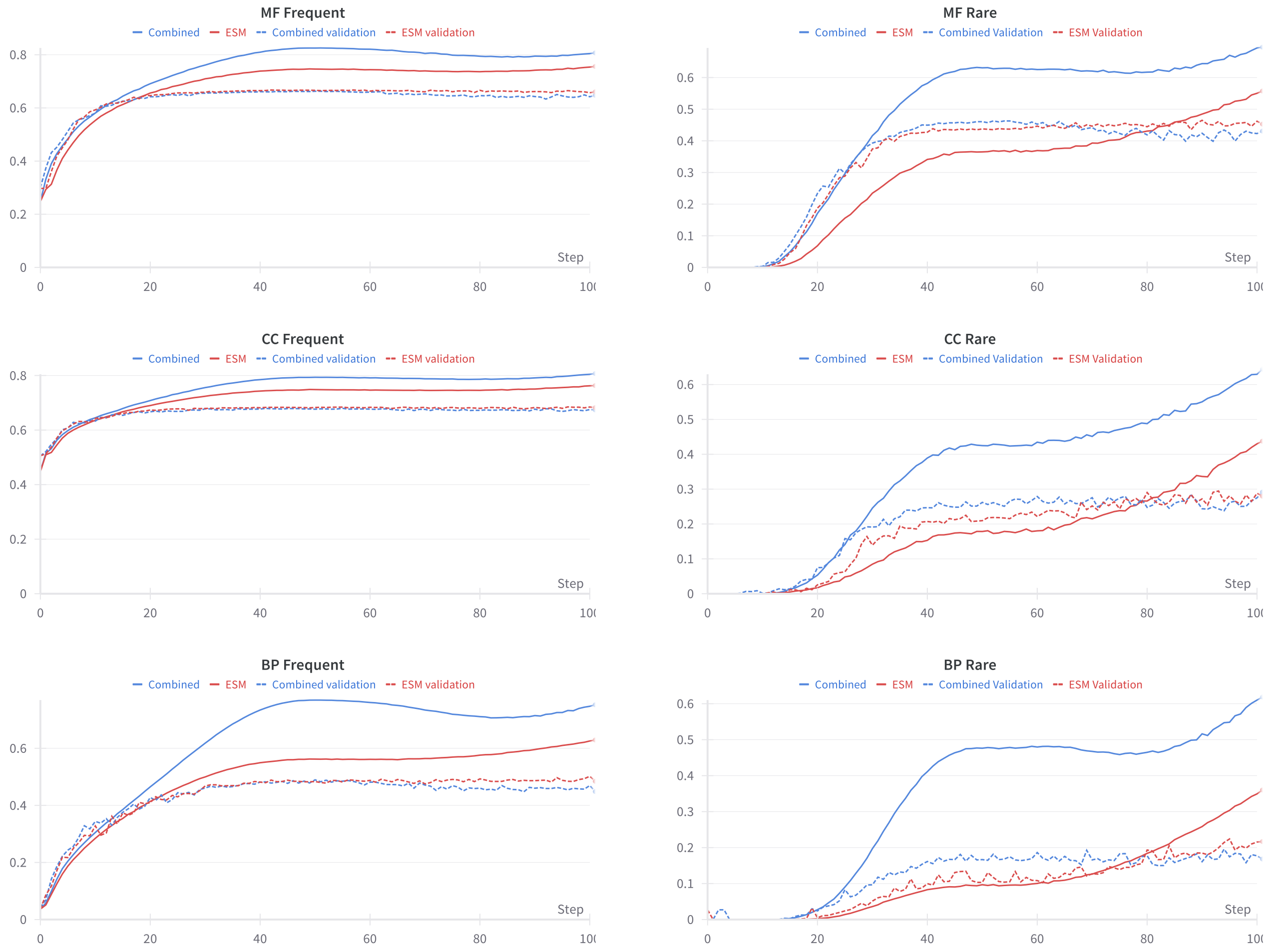

Figure S1: The training and validation curves for TransFew (in red) and TransFew + InterPro + MSA (called Combined). The sub-figures on the left are for the GO terms with annotation frequency greater than or equal to 30 (left) and the sub-figures on the right are for rare GO terms with annotation frequency with less than 30. Throughout the training process (solid lines), TransFew + InterPro + MSA consistently fits the training data better than TransFew, but on the validation data (dashed lines) Transfew performs better.

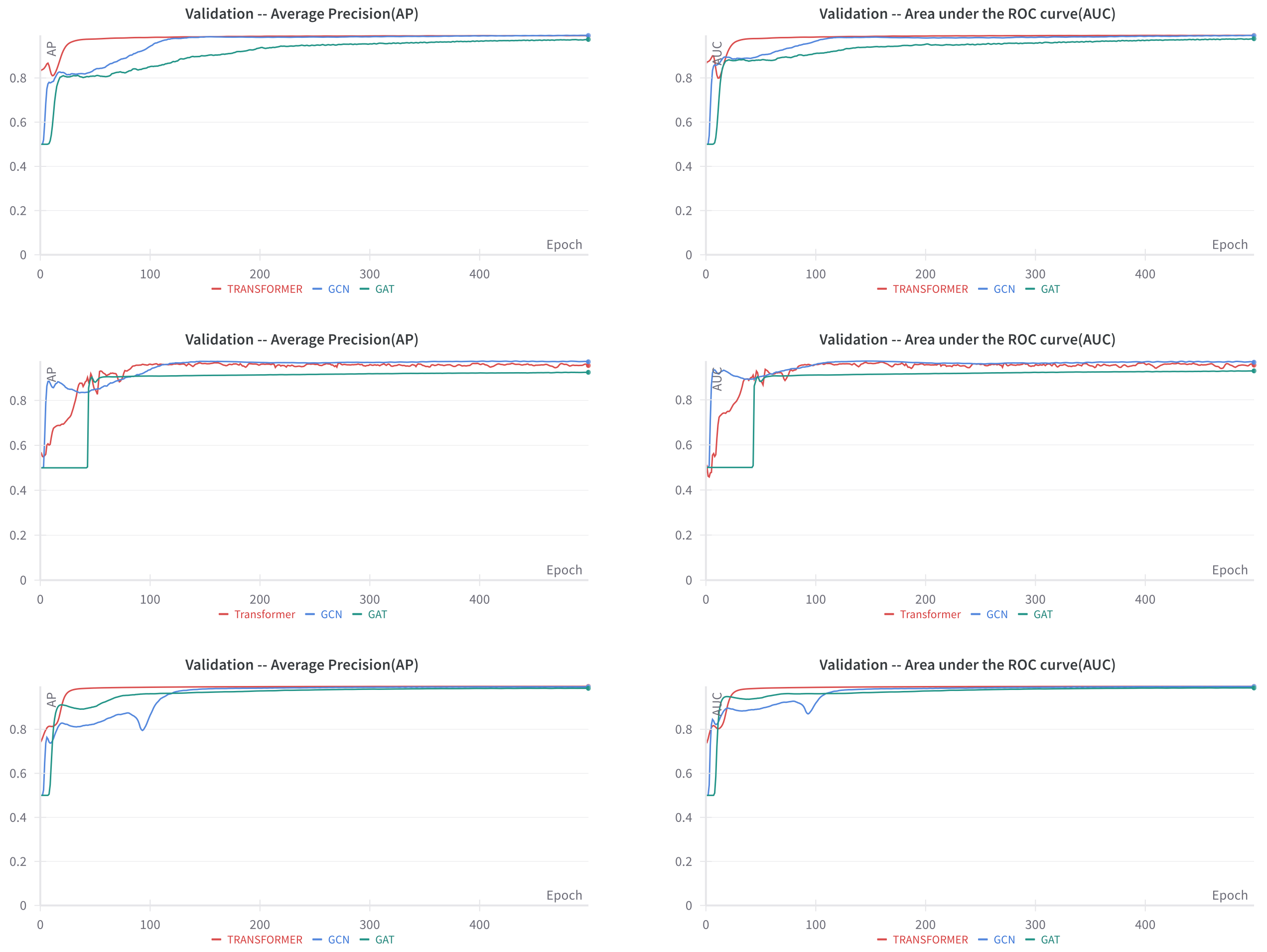

Figure S2: The Average Precision (AP) and Area under the ROC curve (AUC) of three graph neural network-based auto-encoders for three GO categories (molecular function (top), cellular component (middle), and biological process (bottom)). The three encoding architectures have similar performance..

Supplementary Note 5: Evaluation Metrics

In this work, we use the three CAFA[4, 20] evaluation metrics: F_{max}, S_{min}, weighted F_{max} and the area under the precision-recall curve (AUPR) to evaluate protein function predictions, which are defined as follows.

- **Precision**

$$\mathrm{pr}(\tau) = \frac{1}{m(\tau)} \sum_{i=1}^{m(\tau)} \frac{\sum_f \mathbb{I}(f \in P_i(\tau) \wedge f \in T_i)}{\sum_f \mathbb{I}(f \in P_i(\tau))}$$

- **Recall**

$$\mathrm{rc}(\tau) = \frac{1}{n_e} \sum_{i=1}^{n_e} \frac{\sum_f \mathbb{I}(f \in P_i(\tau) \wedge f \in T_i)}{\sum_f \mathbb{I}(f \in T_i)}$$

- **$\mathbf{F_1}$ Score**

$$F_{1(\tau)} = 2 \times \frac{\mathrm{pr}(\tau) \times \mathrm{rc}(\tau)}{\mathrm{pr}(\tau) + \mathrm{rc}(\tau)}$$

- **Maximum $\mathbf{F_1}$ Score**

$$F_{\max} = \max_{\tau} \left(F_1(\tau)\right)$$

  where f is a term, $P_i(\tau)$ is the set of predictions, T_i denotes the corresponding ground-truth, i represents the protein sequence under consideration, and τ is the decision threshold. $m(\tau)$ is the number of proteins sequences with at least one predicted score greater than or equal to the decision threshold τ, $\mathbb{I}(\cdot)$ is an indicator function, and n_e is the number of proteins in the test set for a particular test study.

- **Information Content** (ic) of term f is computed as:

$$\mathrm{IC}(f) = \log_2 \frac{1}{\Pr(f|P(f))}$$

- **Weighted precision**:

$$\mathrm{wpr}(\tau) = \frac{1}{m(\tau)} \sum_{i=1}^{m(\tau)} \frac{\sum_f ic(f) \cdot \mathbb{I}(f \in P_i(\tau) \wedge T_i(\tau))}{\sum_f ic(f) \cdot \mathbb{I}(f \in P_i(\tau))}$$

- **Weighted Recall**:

$$\text{wrc}(\tau) = \frac{1}{n_e}\sum_{i=1}^{n_e}\frac{\sum_f ic(f)\cdot \mathbb{I}(f \in P_i(\tau) \wedge T_i(\tau))}{\sum_f ic(f)\cdot \mathbb{I}(f \in T_i(\tau))}$$

Here, $\Pr(f|P(f))$ represents the probability that term f in the ontology is associated with a protein given that all of its parents are associated.

- **Remaining Uncertainty**

$$ru(\tau) = \frac{1}{ne}\sum_{i=1}^{ne}\sum_{f} ic(f)\cdot \mathbb{I}(f \notin P_i(\tau) \wedge f \in Ti)$$

- **Missing Information**

$$mi(\tau) = \frac{1}{ne}\sum_{i=1}^{ne}\sum_{f} ic(f)\cdot \mathbb{I}(f \in P_i(\tau) \wedge f \notin Ti)$$

- S_{min}

$$S_{min} = \min_{\tau}\sqrt{ru(\tau)^2 + mi(\tau)^2}, \tau$$

- **Area under precision recall curve (AUPR)**

$$\text{AUPR} = \int_0^1 \text{Precision}(R)\, dR$$

where Precision(R) represents the precision at a given recall level (R).

Supplementary Note 6: Experimental set-up

The study leveraged UniProtKB/Swiss-Prot protein data released by November 2022, using 90% for training and 10% for validation. GO terms were expanded using strong evidence codes. An independent test set, named Test_all, comprises proteins annotated up to December 2023. A subset, Test_novel, includes proteins with less than 30% sequence identity to the training set to evaluate model generalization. To construct the test set, the CAFA benchmark generator was employed alongside two Gene Association Format (GAF) files: goa_uniprot_all.gaf.212 and goa_uniprot_all.gaf.218. Swiss-Prot entries were extracted to form Test_all utilizing the corresponding Gene Product Infor-

Table S2: The number of proteins in training, validation, Test_all, and Test_novel datasets for each of the three GO categories (BP, MF, and CC). Additionally, we show the number of proteins in the Trembl.

**Ontology**	**Train**	**Validation**	**Test_all**	**Test_novel**	**Trembl**
BP	82,989	9,221	1101	883	5265
MF	70,773	7,864	1112	858	1745
CC	83,620	9,292	782	622	2974

mation file (GPI). Following this, sequences in Test_all with over 30% identity to the training data were filtered out using MMseqs, resulting in Test_novel. Dataset statistics are provided in Table S2. Evaluation was performed using the CAFA-evaluator[11], with the best score reported for each metric (Fmax, weighted Fmax, and S_{min}). Additionally, we compute the AUPR for each method using the trapezoidal rule on the precision and recall values provided by the CAFA-evaluator, without interpolating the precision-recall curve at the extremes. For Information Accretion, the InformationAccretion repository[10] was utilized.

We compared TransFew with six baseline methods, namely Naive, DiamondBLAST, Tale, NetGO 3.0, DeepGO-SE, and SPROF-GO. Here's a concise overview of each method:

Naive: The Naive method simply uses the frequency of Gene Ontology (GO) terms in the training dataset to make predictions.

DiamondBLAST: Based on sequence similarity scores obtained through BLAST, it identifies similar sequences from the training set and transfers annotations from the most similar ones [2, 7].

Tale: A transformer-based method integrating protein sequence and label features to predict protein function by jointly embedding sequence and hierarchical label information [3]. Predictions for Tale were generated by downloading the code from GitHub and running it locally.

NetGO 3.0: An ensemble method combining outputs from seven individual function prediction methods using various input sources, including Naive prediction, BLAST-KNN, LR-3mer, LR-InterPro, Net-KNN, LR-Text, and LR-ESM [8, 13, 15, 17, 18]. Test predictions were obtained through the NetGO3 web server

SPROF-GO: An alignment-free method employing a pre-trained protein language model to extract informative sequence embeddings. It utilizes self-attention pooling to focus on crucial residues

and integrates homology information using a label diffusion algorithm [19]. Test predictions were obtained through the provided web server for SPROF-GO.

DeepGO-SE: Utilizes a pre-trained large protein language model combined with GO background knowledge and protein-protein interactions (PPIs) to make accurate predictions about protein functions [6]. Predictions for DeepGO-SE were generated by cloning and running the tool locally.

Supplementary Note 7: Experiment on Label Embedding

To test if using the label encoder improved prediction accuracy, we replaced the embedding of the label encoder with a random matrix for each GO partition group to check how the performance was changed. Specifically, for Partition x, where $x = 1,2$ for MF and CC, and $x = 1,2,3$ for BP, the embedding of all GO terms in the partition/group was substituted by a random matrix. For instance, for Partition 1 in CC, we replaced the label encoder for all 873 GO terms with a random matrix. The results after replacing the GO term encoding with random matrix are compared with TransFew of employing the label encoder are shown in the table below. The results show that using the label encoder to generate the representation for GO terms improves the prediction accuracy across the board.

Table S3: The performance of different implementations of TransFew on the Test_all dataset that use a random matrix to replace the embedding of GO terms in a partition generated by the label encoder in comparison with the final TransFew that uses the label encoder.

**Methods**	F_{max} (↑)			WF_{max} (↑)			$AUPR$ (↑)			S_{min} (↓)		
	**CC**	**MF**	**BP**	**CC**	**MF**	**BP**	**CC**	**MF**	**BP**	**CC**	**MF**	**BP**
Partition 1	0.682	0.437	0.414	0.544	0.369	0.366	0.732	0.5858	0.382	8.047	17.948	20.166
Partition 2	0.610	0.570	0.400	0.427	0.455	0.352	0.651	0.5422	0.353	8.826	7.732	20.412
Partition 3	NA	NA	0.418	NA	NA	0.370	NA	NA	0.382	NA	NA	19.875
TransFew	**0.726**	**0.665**	**0.449**	**0.611**	**0.586**	**0.407**	**0.793**	**0.687**	**0.439**	**6.694**	**6.385**	**19.353**

References

Brody, S., Alon, U., & Yahav, E. (2021). How attentive are graph attention networks? *arXiv preprint arXiv:2105.14491*.

Buchfink, B., Xie, C., & Huson, D. H. (2015). Fast and sensitive protein alignment using diamond. *Nature methods*, *12*(1), 59–60.

Cao, Y., & Shen, Y. (2021). Tale: Transformer-based protein function annotation with joint sequence–label embedding. *Bioinformatics*, *37*(18), 2825–2833.

Jiang, Y., Oron, T. R., Clark, W. T., Bankapur, A. R., D'Andrea, D., Lepore, R., … others (2016). An expanded evaluation of protein function prediction methods shows an improvement in accuracy. *Genome biology*, *17*(1), 1–19.

Kipf, T. N., & Welling, M. (2017). Semi-supervised classification with graph convolutional networks. In *International conference on learning representations (iclr).*

Kulmanov, M., Guzmán-Vega, F. J., Duek Roggli, P., Lane, L., Arold, S. T., & Hoehndorf, R. (2023). Deepgo-se: Protein function prediction as approximate semantic entailment. *bioRxiv*, 2023–09.

Kulmanov, M., & Hoehndorf, R. (2020). Deepgoplus: improved protein function prediction from sequence. *Bioinformatics*, *36*(2), 422–429.

Le, Q., & Mikolov, T. (2014). Distributed representations of sentences and documents. In *International conference on machine learning* (pp. 1188–1196).

Mirdita, M., von den Driesch, L., Galiez, C., Martin, M. J., Söding, J., & Steinegger, M. (2016, 11). Uniclust databases of clustered and deeply annotated protein sequences and alignments. *Nucleic Acids Research*, *45*(D1), D170-D176. Retrieved from https://doi.org/10.1093/nar/gkw1081 doi: 10.1093/nar/gkw1081

Paolis, C. D. P. D. (2023). *Informationaccretion.* https://github.com/claradepaolis/InformationAccretion. (Last Accessed: 2024-05-14)

Piovesan, D., Zago, D., Joshi, P., De Paolis Kaluza, M. C., Mehdiabadi, M., Ramola, R., ... others (2024). Cafa-evaluator: a python tool for benchmarking ontological classification methods. *Bioinformatics Advances*, *4*(1), vbae043.

Rao, R., Liu, J., Verkuil, R., Meier, J., Canny, J. F., Abbeel, P., ... Rives, A. (2021). Msa transformer. *bioRxiv*. Retrieved from https://www.biorxiv.org/content/10.1101/2021.02.12.430858v1 doi: 10.1101/2021.02.12.430858

Rives, A., Meier, J., Sercu, T., Goyal, S., Lin, Z., Liu, J., ... Fergus, R. (2019). Biological structure and function emerge from scaling unsupervised learning to 250 million protein sequences. *PNAS*. Retrieved from https://www.biorxiv.org/content/10.1101/622803v4 doi: 10.1101/622803

Shi, Y., Huang, Z., Feng, S., Zhong, H., Wang, W., & Sun, Y. (2020). Masked label prediction: Unified message passing model for semi-supervised classification. *arXiv preprint arXiv:2009.03509*.

Szklarczyk, D., Franceschini, A., Wyder, S., Forslund, K., Heller, D., Huerta-Cepas, J., ... others (2015). String v10: protein–protein interaction networks, integrated over the tree of life. *Nucleic acids research*, *43*(D1), D447–D452.

Veličković, P., Cucurull, G., Casanova, A., Romero, A., Lio, P., & Bengio, Y. (2017). Graph attention networks. *arXiv preprint arXiv:1710.10903*.

Wang, S., You, R., Liu, Y., Xiong, Y., & Zhu, S. (2023). Netgo 3.0: Protein language model improves large-scale functional annotations. *Genomics, Proteomics & Bioinformatics*.

Yao, S., You, R., Wang, S., Xiong, Y., Huang, X., & Zhu, S. (2021). Netgo 2.0: improving large-scale protein function prediction with massive sequence, text, domain, family and network information. *Nucleic acids research*, *49*(W1), W469–W475.

Yuan, Q., Xie, J., Xie, J., Zhao, H., & Yang, Y. (2023). Fast and accurate protein function prediction from sequence through pretrained language model and homology-based label diffusion. *Briefings in bioinformatics*, *24*(3), bbad117.

Zhou, N., Jiang, Y., Bergquist, T. R., Lee, A. J., Kacsoh, B. Z., Crocker, A. W., … others (2019). The cafa challenge reports improved protein function prediction and new functional annotations for hundreds of genes through experimental screens. *Genome biology*, *20*(1), 1–23.
